# Calcitonin Gene-Related Peptide Antagonists as a Savior in Episodic and Chronic Migraine: A Review

**DOI:** 10.7759/cureus.8711

**Published:** 2020-06-20

**Authors:** Hira Pervez, Lavina Khemani, Mahrukh A Khan, Ahmed M Seedat, FNU Roshan

**Affiliations:** 1 Internal Medicine/Cardiology, Dow University of Health Sciences, Karachi, PAK; 2 Internal Medicine, Dow University of Health Sciences, Karachi, PAK; 3 Family Medicine, Dow University of Health Sciences, Karachi, PAK; 4 Internal Medicine, Jinnah Sindh Medical University, Karachi, PAK

**Keywords:** migraine, migraine disorder, monoclonal antibodies, chronic migraine, episodic migraine, galcanezumab, fremanezumab, erenumab, eptinezumab, gepants

## Abstract

Headaches due to migraine are the second leading cause of disability in the world. Migraine can be classified as episodic migraine (EM) and chronic migraine (CM). The course of the disease starts from an aura followed by 4-72 hours of bouts of throbbing, mostly unilateral headache associated with nausea, photo/phonophobia with/without neurological deficit. The pathophysiology of migraine remains debatable and many drugs are used to help control migraine attacks with little or no benefit. However, patient compliance remains a reason for over and underdosing of these medications. The calcitonin gene-related peptide (CGRP), a vasoactive peptide is known to contribute to the disease course. Much work is done on antagonizing the receptor or the molecule itself. For this purpose, genetically engineered monoclonal antibodies are being utilized for long-term reduction in morbidity and prevention of migraine headaches. The four to name are: galcanezumab, fremanezumab, eptinezumab, and erenumab. The purpose of this review is to shed light on the use of these monoclonal antibodies, completed and recruiting trials, and the role of these medications in the prevention of not only EM and CM but also in medication overuse headaches.

## Introduction and background

Migraine remains a challenging disease throughout the world affecting around 12% of the population [1]. Migraine disorder affects individuals with variable symptoms. It may manifest as an aura followed by the headache which can be unilateral or bilateral and may be associated with nausea, photo, and/or phonophobia [2]. It is known to be the second leading cause of disability in the young and working population. Acute migraine attacks can progress and lead to chronic migraine (CM). It is estimated that around 2.5% of patients who present at first with episodic migraine (EM) can progress to debilitating chronic and recurrent migraine headaches. This can be very crucial for individuals who have a daily routine to follow, leading to unusual absences from work and can also lead to psychiatric as well as diseases involving other organs. Many treatment modalities ranging from abortive to preventive therapies are instituted to reduce the morbidities associated with this disease. However, treatment failure in the form of over/under-dosing is seen due to these novel drugs. To overcome this hurdle, drug manufacturers are struggling to discover medications that can be administered once or twice a month by parenteral means increasing their bioavailability and patient compliance. Our review discusses the cardinal drugs antagonizing the vasoactive peptide responsible for the pathogenesis of migraine headaches. This peptide is termed as calcitonin gene-related peptide (CGRP). The drugs used to counteract the molecule and/or its receptor are genetically engineered monoclonal antibodies, most of which are officially approved by the Food and Drug Administration [3].

A detailed literature search using electronic databases including PubMed, Clinicaltrials.gov, and Google Scholar was conducted. “Migraine”, “chronic migraine disorder”, "calcitonin gene-related peptide”, “monoclonal antibodies” were used as MeSH terms for data search. The reviewed articles were within five years, preferably in the English language. Our initial search included 53 articles from PubMed, 12,300 from Google Scholar, and 24 studies in clinicaltrials.gov. All articles focusing on CM and EM, and all completed trials were included while articles with acute attacks of migraine, co-morbid conditions (diabetes, cardiovascular diseases, etc), and migraine-like conditions were excluded. Further refining the search on the inclusion and exclusion criteria dropped the final articles to 20 from PubMed, 230 from Google Scholar, and 13 completed trials from clinicaltrials.gov. References cited from the initial search were used to obtain further literature. Currently recruiting and completed trials were included to increase the authenticity of the literature. Articles including conditions other than migraine, migraine in menstruation, co-morbidities associated with migraine, and other articles that lay beyond our study were excluded. A detailed screening and thorough read of all the included full-text articles were also conducted.

## Review

Headaches due to migraine disorder can present in a variety of ways. Pain as a symptom can compromise an individual's daily routine in general. However, pain originating due to hypersensitivity of the nerves can be severely debilitating. The variable presentation of this neurological disorder specifies its complexity, from mild to severe pulsating attacks of headache associated with other symptoms. Much work is done and a lot is available in the literature regarding the disease course of migraine headaches. A study on the global burden of this disease conducted in 2016 declared migraine headaches as the leading cause of disability in the young, working population, with a prevalence of 15%-18% and is ranked as the sixth prevalent disease worldwide [4-5]. This disease is known to follow a staged course ranging from premonitory stage to aura followed by bouts of headache and culminating into a post-dromal phase very similar to the premonitory stage. The aura here manifests as unilateral, reversible episodes of single or multiple neurological deficits, progressing over minutes to hours. This is associated with photo and/or phonophobia with nausea and the cardinal symptom of headaches lasting between 4 and 72 hours duration [6]. As the disease comes in bouts, many patients may present frequently in a week or up to 1.5 times a month.

As the presentation of this disease is variable, so is its pathophysiology. Individuals harboring this ailment are seen to possess a variety of genetic makeup making it a multifactorial genetic disorder. This, in turn, can impact different structures ranging from neurons to the blood vessels in close proximity to these neurons. Syndromes that have migraine or migraine-like symptoms also have the involvement of different genes. The commonest ones to highlight are familial advanced sleep-phase syndrome, a familial hemiplegic migraine, and cerebral autosomal dominant arteriopathy with subcortical infarcts and leukoencephalopathy [7].

Migraine headaches are classified depending upon the days per month an individual is affected. They can be EM with less than 15 days and CM with at least 15 days of which eight days or more are migraine days. This front of the disease can be extremely debilitating posing such individuals at high risk with medication overuse headache, increasing disease severity than reducing it [8].

Mechanism of migraine

As the mechanism of migraine remains unclear, an understanding of the pathology of the disease may help investigators to ascertain the therapeutic options and pose a meaningful influence on the impact of the disease. A study by Goadsby et al. highlighted the perspective of nervous sensitization to pain in migraine. The vessels embedding the dura mater are innervated by the nerve endings of the trigeminal nerve and dorsal root ganglion. Several vasoactive peptides similar to the ones secreted extracranially like serotonin, nor-adrenaline, acetylcholine, etc. are released by these nerve endings leading to vasodilation of the vasculature involved in the disease process. CGRP is one such neuropeptide that surges during attacks of migraine and remains consistently elevated during the disease course [6, 9]. Acute attacks of migraine are usually targeted with the use of analgesics. In the preventive arm of the therapeutic approach, significant work is done to improve the quality of life of patients encountering this ailment. To better control CM, many drugs have been used over decades that were recommended for preventing EM at first. According to the Health and Human Services, drugs like topiramate, valproate, serotonin receptor antagonists are seen to work well [10]. The challenge to this approach is the use of "triptans"- novel drugs for EM. These drugs target the pathology of the disease and lead to vasoconstriction. However, certain limitations have decayed their role in the chronicity of the disease; these include adverse cardiovascular outcomes and a higher risk of developing drug-induced headaches if taken longer. Literature suggests that the use of acute headache medications has led EM to transit to CM. This is due to the lack of adequate response to these drugs in different patients, increasing the recurrence of migraine attacks [11-12].

What is calcitonin gene-related peptide?

Calcitonin gene-related peptide is a vasoactive neuropeptide released by the nociceptive neuronal nerve endings. On the genetic level locus heterogeneity produces different isoforms of CGRP. It is coded alongside calcitonin on chromosome number 11 by the CALC I gene and is produced by alternative splicing in other specific tissues. Likewise, the CALC II gene located at a different locus on the same chromosome is responsible for the production of beta CGRP. Alpha CGRP, the main target for CGRP antagonists, is a 37 amino acid sequence isoform that is exclusively found in the central nervous system [11]. This neuropeptide is responsible for inflammatory reactions leading to neurogenic inflammation. As we know, at the onset of a stimulus to the nerve fibers, action potentials are generated. The transient receptor potential that is fired by the dural afferent sensory nerve fibers leads to the release of molecules like CGRP [13]. After the peptide is released it binds to its respective receptor and is internalized using dynamin/clathrin complexed with beta-arrestin and is recycled back to the plasma membrane. The receptor comprises a transmembrane receptor component; calcitonin-like receptor, receptor activity modifying protein 1, and receptor component protein. A hypothesis for consumption and reuptake system suggests that various enzymes play a role in the metabolism of the molecule like; neprilysin, endothelin-converting enzyme 1, and insulin-degrading enzyme. However, receptor stimulation by CGRP can chronically lead to its desensitization and degradation [11, 14-15]. CGRP involvement in the whole disease process was described by Edvinsson approximately 30 years back [11]. The presence of CGRP in the central, as well as the peripheral nervous system and its defined role in the pathogenesis of migraine (retrograde pathway from the ganglion of the fifth cranial nerve to the brain after internalization), is now able to guide researchers towards the role of agents targeting this neuropeptide. This neuromodulator and its receptor are targeted using immunological techniques utilizing monoclonal antibodies. At first, "gepants", small molecules were developed. These are competitively bound to the CGRP receptor to antagonize its function. They were used in the prophylactic treatment of migraine. However, the toxicity profile did not allow these drugs to pass on to the general public [16].

Monoclonal Antibodies Against CGRP

To combat the morbidity associated with migraine and the shortcomings with various therapeutic modalities, a new set of drugs in the form of monoclonal antibodies acting against CGRP is at an escalating trend. These antibodies can block both the CGRP and/or its receptor. Several clinical trials studied the role of these monoclonal antibodies in preventing migraine attacks. To date, four novel monoclonal antibodies are in use to fight against EM as well as CM. Of these four, three function against the CGRP molecule while one is known to antagonize the CGRP receptor. To fully understand their mechanism of action, a view on their kinetics and dynamics is essential.

These monoclonal antibodies are produced by hybridomas (cells producing the same type of antibodies). The "zumabs" are humanized and functionally grafted to fit the human "Fab" zone and are formed from the complementarity determining regions (CDR) from various animal models. Fremanezumab and galcanezumab are grafted from CDR from the mouse model while eptinezumab is grafted from the rabbit. The only monoclonal antibody, which is human is erenumab [17]. These biologic agents have unique kinetics and dynamics which are slightly different from the novel chemicals. In addition to their pharmacokinetics, many other aspects are considered essential when these drugs are delivered to patients. Most of these large agents have a molecular weight of approximately 150-kilo dalton [18]. This raises questions on their mode of administration, location of the action, and their plasma half-life. Considering their composition and large size their ability to penetrate the blood-brain barrier (BBB) is significantly limited. This points to the fact that the mechanism of action of these medications is not directed to the central nervous system. Moreover, they are seen to efficiently work in areas not directly protected by the BBB. These structures may include vascular zones, the sensory ganglion of the fifth cranial nerve, and the structures surrounding the ventricles. In addition to this, the drugs have comparatively longer half-lives and play a role in the prophylactic management of migraine. The route of administration is also selected as parenteral as these drugs take a long time to achieve their maximum concentration in the blood [16]. Compliance and tolerability of medication use remain the most common reason for treatment failure. However, due to the longer half-lives of these monoclonal antibodies, they do not require daily dosing and can be administered every month [19].

Several completed and a few ongoing clinical trials have proved the efficacy of these monoclonal antibodies in the prevention as well as treatment of both EM and CM.

Galcanezumab

Galcanezumab is an IgG4 antibody that is administered subcutaneously [20]. This humanized monoclonal antibody was tested in trials for both EM as well as CM. These changes were seen in randomized controlled EVOLVE trials. In both EVOLVE 1 AND 2, the primary end-point was a reduction in monthly migraine days (MMDs). EVOLVE 1 was a phase 3 double-blinded, placebo-controlled randomized clinical trial in which two doses of galcanezumab in the potency of 120 and 240 mg were used versus placebo to check the efficacy of the drug. Both doses were administered subcutaneously once a month for six months. There was a significant reduction in the MMDs of 4.7 and 4.6 days for each of the two doses respectively as compared to the 2.8 days for placebo [21]. Similarly, EVOLVE 2 looked into both the efficacy and safety profile of galcanezumab use in patients with EM. The same dosage of this drug was administered subcutaneously once-monthly dosing for six months to all the participants. A gross reduction in mean MMDs of 4.3 days for 120 mg and 4.2 days for 240 mg was seen as compared to placebo [22]. CM remains a reason for recurrent attacks. A double-blinded, placebo-controlled, phase 3 randomized clinical trial suggested the use of galcanezumab for the prevention of CM. In this study, both the doses of galcanezumab were seen to be efficacious with a reduction in the MMDs of 4.8 and 4.6 days for 120 and 240 mg respectively, as compared to 2.7 MMDs for placebo [12].

Fremanezumab

Fremanezumab remains yet another remedy for the treatment of migraine and its variable presentation. It is a humanized IgG2k monoclonal antibody, selectively antagonizing the CGRP molecule [20]. Different clinical trials have been undertaken to modify treatment strategies to minimize the attacks of headaches associated with migraine. For this reason, the drug was investigated in a double-blind, multi-centered, randomized (HALO) clinical trial. The HALO trial looked into the efficacy, tolerability, and long-term safety profile of fremanezumab. This study suggested that MMDs of EM were reduced to 3.7 days at 225 mg of the drug at 12 weeks and 3.4 days at 675 mg given for the same duration of time, while there was a reduction of MMD by 2.2 days in the placebo arm of the study [23]. Many medications have been used to prevent as well as treat migraine headache disorder. As discussed above, these drugs have their limitations and lead to treatment failure. FOCUS trial was conducted to evaluate the efficacy, safety, and tolerability of fremanezumab in patients who were resistant to medications in improving migraine-related headaches. These patients exhibited a clinically significant improvement in CM as well as EM, with a baseline reduction of MMDs of 3.5 days as compared to placebo in the quarterly and/or monthly dosing. This study proved that fremanezumab can be used in the prevention of various migraine attacks [24]. To reduce morbidity in patients with migraine, a 12-week, phase 3, double-blind, placebo-controlled, randomized clinical trial was conducted to evaluate the efficacy, safety, and side-effect profile of fremanezumab administered in two subcutaneous doses for the prevention of CM. Two dosing regimen with 675 mg single dose was given quarterly versus placebo at four and eight weeks. The other regimen included monthly dosing of 675 and 225 mg at four and eight weeks versus placebo. It was concluded that there was a significant reduction in the frequency of headaches with fremanezumab [5].​​​​​​​

Eptinezumab

Eptinezumab, the only known monoclonal antibody that is administered intravenously is a genetically engineered IgG1k humanized monoclonal antibody that has been seen to work effectively in several clinical trials over the globe to provide relief from this debilitating ailment [20]. Few ongoing trials to mention are "RELIEF" and another that is currently being done on participants in Japan [25-26].

To assess the effectiveness of the drug to prevent frequency in EM, participants were recruited in two trials PROMISE 1 and 2. These two placebo-controlled, double-blind, phase 3 randomized clinical trials were seen to evaluate the efficacy and safety profile of eptinezumab. The drug was randomly administered to study participants in 30, 100, and 300 mg potency versus placebo for 12 weeks. A mean-reduction of 8.6 MMDs was observed in the treatment group. Besides, preventive effects were seen following a single intravenous dose of eptinezumab [27]. PROMISE 2 was shown to evaluate the efficacy and safety of eptinezumab in the prevention of CM. Two doses of 100 and 300 mg were administered in study participants versus placebo. The study showed promising results with a reduction in MMDs from 16.1 to 8.5 days in the 100 mg group, 7.9 MMDs in the 300 mg group versus 10.5 MMDs in the placebo group [28]. An open-label "PREVAIL" trial was also conducted to study the safety profile of eptinezumab. The study included 128 participants in a single group assignment. Treatment-emergent adverse events for 104 weeks were monitored. The primary outcomes like changes in the electrocardiogram, lab values, vital signs, and suicidal ideation were tracked throughout the study [29]. The "RELIEF" trial is a double-blind, placebo-controlled, phase-3 randomized clinical trial, currently recruiting participants and is seen to evaluate the efficacy and safety of eptinezumab when administered intravenously during acute migraine attacks versus placebo. Participants in the experimental arm are receiving a single dose of 100 mg eptinezumab versus a placebo. Headache pain freedom time is observed as the primary end-point [25]. Healthy Japanese participants are currently being recruited in a double-blind, placebo-controlled, single-ascending-dose, randomized clinical trial. This particular study is conducted to investigate the pharmacokinetics, safety, and tolerability of eptinezumab [26].

Erenumab

Among the four CGRP antagonists for the prevention and treatment of migraine, erenumab is the only human IgG2λ, administered subcutaneously and acting on the CGRP canonical receptor [19-20]. Many clinical trials evaluating the molecule are currently under study and a few are completed. The drug is seen to be effective in EM as well as CM. For this purpose, a phase-2 randomized controlled trial was conducted to evaluate the efficacy and safety of erenumab in the prevention of EM. Three doses of 7, 21, and 70 mg were administered subcutaneously versus placebo. The MMDs with 70 mg dose was found to be -3.4 days as compared with -2.3 days of placebo. The rest of the two doses did not have a significant difference in MMDs when compared with placebo [30]. Much work is done to prevent the attacks of migraine. Another phase-3 double-blind, placebo-controlled, randomized clinical trial was conducted to evaluate the efficacy and safety of erenumab versus placebo. A drug dose of 70 mg was used for this purpose. There was a reduction in the MMDs of 2.9 days as compared to 1.8 days of placebo [31]. Erenumab, in the potency of 70 and 140 mg, administered monthly for six months was used in a phase-III clinical trial "STRIVE". This trial investigated the effectiveness of the drug as a preventive source in EM [32]. A new trial namely "EMPOwER" is being conducted to evaluate the efficacy and safety of erenumab in preventing EM headaches in the adult population. It is a single cohort, double-blind, placebo-controlled, randomized clinical trial. The primary end-points are reduction in the MMDs at three-months from the start of the treatment versus placebo. The drug is administered in a pre-filled syringe [33].

Medication Overuse Headache

Medication compliance remains a debate for managing chronic debilitating illnesses worldwide. Patients compel to use their prescribed medications for quick relief of such conditions. As stated earlier, migraine remains the second most common cause of disability in the world [3]. In such a scenario, drug over and underdosing can create discrepancies for both the patients and their physicians. One such condition is medication overuse headache. It is seen to occur in patients with CM using a variety of medications. Patients may remain dissatisfied with the medication dosing and may escalate either the dose of the drug or its frequency. In such a condition, proper awareness and guidance to reduce any such medications are provided to the patient. Immediate cessation or gradual tapering of the drug and prevention of such bouts is also deemed important. In this context, the prevention of this condition is also achieved using CGRP monoclonal antibodies and botulinum toxin type A [34].

## Conclusions

Migraine headache is a complex disorder of the central as well as the peripheral nervous system. The pathophysiology of this disease is as complicated as the ailment itself. Due to this, different treatment options are incorporated to deliver maximum benefits. However, the novel drugs used in the treatment of migraine headaches have shown limited cure. Patient compliance along with adverse effect profiles can be some of the reasons. The advent of the CGRP antagonists has opened a new avenue in the treatment as well as prevention of the variable symptoms of migraine disorder. The route of administration, the dosing, and the reduction in the disabling symptoms are curtailing the treatment guidelines for CM as well as EM.
